# Increased Mevalonate Production Using Engineered Citrate Synthase and Phosphofructokinase Variants of *Escherichia coli*

**DOI:** 10.1002/bit.28902

**Published:** 2024-12-09

**Authors:** Jeffrey K. Dodelin, Abigail E. Rose, Hemshikha Rajpurohit, Mark A. Eiteman

**Affiliations:** ^1^ Department of Microbiology University of Georgia Athens Georgia USA; ^2^ School of Chemical, Materials and Biomedical Engineering University of Georgia Athens Georgia USA

**Keywords:** acetyl‐CoA, batch process, citrate synthase, GltA, isoprenoids, mevalonate, nitrogen‐limitation, PfkA

## Abstract

Mevalonate is a biochemical precursor to a wide range of isoprenoids. The mevalonate pathway uses three moles of acetyl‐CoA, and therefore native pathways which metabolize acetyl‐CoA compete with mevalonate synthesis. Moreover, the final step in mevalonate formation, mediated by hydroxymethylglutaryl‐CoA reductase, requires NADPH as a co‐substrate. This study focuses on chromosomal modification of citrate synthase (GltA) involved in acetyl‐CoA utilization and phosphofructokinase (PfkA) involved in NADPH formation to increase the yield and productivity of mevalonate in *Escherichia coli* overexpressing the three genes of the heterologous mevalonate pathway. Nine GltA variants were compared for mevalonate production with the Δ*gltA* knockout and the wild‐type GltA strain in shake flasks in the absence and presence of casamino acids. In the presence of casamino acids, all variants generated mevalonate at a greater yield than the wild‐type control, but less than the GltA knockout. In the absence of casamino acids, the strain expressing wild‐type GltA generated the greatest yield of mevalonate, while most variants instead accumulated primarily acetate. Using the wild‐type strain and two citrate synthase variants, four phosphofructokinase variants were also compared with the Δ*pfkA* knockout and the wild‐type strain, but PfkA variants generated less mevalonate than the corresponding wild‐type PfkA strain. Controlled processes at the 1‐liter scale comparing five strains demonstrated the inverse relationship between yield and productivity, with the GltA[K167A] variant showing the best balance for the yield (0.20 g/g) and productivity (0.87 g/L h). A nitrogen‐limited process using the GltA[K167A] variant generated 36.9 g/L mevalonate in 31 h at a yield of 0.31 g/g. This study demonstrates that GltA variants offer a means to affect intracellular acetyl‐CoA pools for the generation of acetyl‐CoA derived products and that the acetyl‐CoA pool rather than NADPH availability is the important limiting factor for mevalonate production.

## 1 Introduction

Isoprenoids are among the most abundant and structurally diverse naturally occurring compounds. They are found in all Kingdoms of life and serve a myriad of roles including cell signaling (Zaroubi et al. 2022), fruit maturation (Wenkai et al. 2021), and microbial abatement (Guimarães et al. 2019). Isoprenoids have been exploited widely, from low value commodity feedstocks and fuels to highly valuable specialty chemicals and pharmaceuticals (Schwab, Fuchs, and Huang 2013). There are several sources for isoprenoids including fossil fuels (Mewalal et al. 2017) and plants (Schempp et al. 2018). With greater control of environmental conditions than generally can be achieved with plants, and the ability to use enzymes that mediate highly specific, natural reactions (Leavell, McPhee, and Paddon 2016), microbial formation of isoprenoids has emerged as an attractive platform.

**Figure 1:**
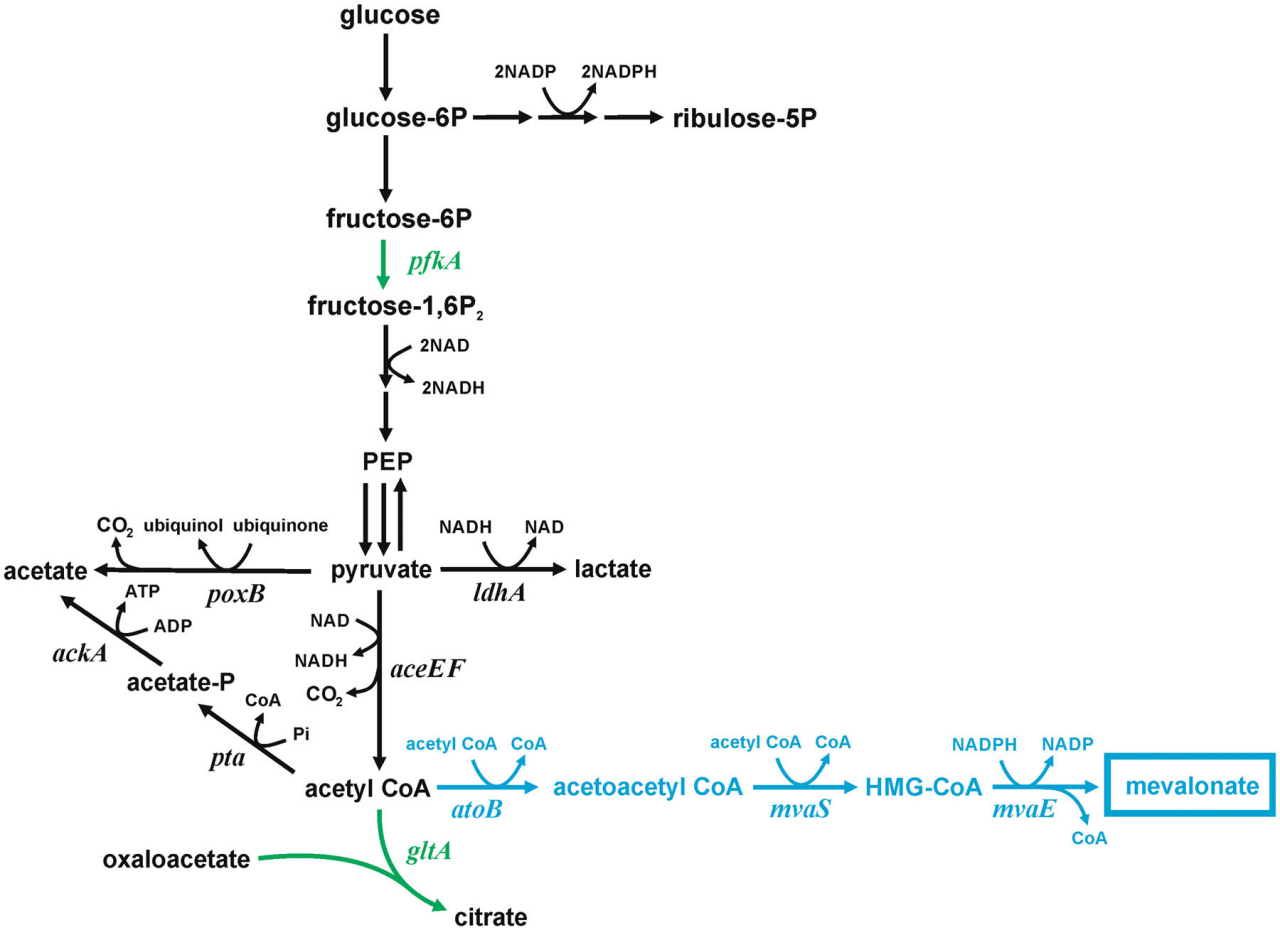
Key biochemical pathways for the conversion of glucose to mevalonate in *E*. *coli*. Fluxes through the citrate synthase and phosphofructokinase (green) were partly curtailed by the introduction of variant alleles coding amino acid substitutions as detailed in the text. The native *E. coli atoB* gene and heterologous *mvaS* and *mvaE* genes from *Lactobacillus casei* (blue) were constitutively expressed on the pMVA1 plasmid (Rugbjerg et al. 2018b).

Mevalonate is a key precursor in a biochemical pathway to isoprenoids. Mevalonate itself is a component in moisturizers (Kuzuyama et al. 2004), and its lactone could be used to treat some forms of statin‐induced myopathy (Yogev et al. 2023). The mevalonate (MVA) pathway, found in eukaryotes and archaea, begins with acetyl‐CoA acetyltransferase (ACAT) condensing two acetyl‐CoA moieties to generate acetoacetyl‐CoA (Figure 1). Hydroxymethylglutaryl‐CoA (HMG‐CoA) synthase (HMGS) subsequently adds a third acetyl‐CoA to form HMG‐CoA, while HMG‐CoA reductase (HMGR) reduces HMG‐CoA with NADPH to mevalonate. For isoprenoid synthesis, mevalonate is further phosphorylated by mevalonate‐5‐kinase and phosphomevalonate kinase to form mevalonate pyrophosphate, which is decarboxylated by mevalonate pyrophosphate decarboxylase to form the isoprenoid building block isopentyl‐5‐pyrophosphate (Lombard and Moreira 2011).

Numerous approaches have been examined to increase the titer and productivity of mevalonate via the MVA pathway in *E. coli*. For example, deletions of nine genes toward unnecessary by‐products increased mevalonate production two‐fold (Kim et al. 2016). Since the final reduction to mevalonate requires NADPH, another general approach to improve mevalonate formation is to alter cellular NADPH generation. For example, careful induction of the *pgi* gene coding glucose‐6‐phosphate isomerase increases the relative flux into the pentose phosphate pathway over the Embden‐Meyerhof‐Parnas pathway and increases mevalonate yield by 25% (Kamata, Toya, and Shimizu 2019). Specific mevalonate productivity and glucose uptake increased nearly twofold in a strain with an E672K substitution in the *rpoB* gene, encoding RNA polymerase subunit B. This mutation increases DNA binding efficiency of RpoB and transcription globally while downregulating other cellular processes like chemotaxis and flagellar development (Rugbjerg, Feist, and Sommer 2018a). Others have focused on limiting the consumption of carbon toward biomass and making more available to mevalonate, by limiting growth using a non‐carbon nutrient. For example, initial sulfate limitation in shake flasks led to an 80% increase in the mevalonate yield from glucose (Li, Jendresen, and Nielsen 2016). Similarly, *E. coli* harboring an inducible mevalonate pathway were first grown aerobically with glucose, then switched to acetate, and gene transcription was induced under aerobic, microaerobic, or anaerobic conditions. In this second phase, aerobic conditions achieved the highest mevalonate titer (7.9 g/L) and productivity (0.13 g/L h). However, mevalonate yield (0.37 g/g_acetate_) was the greatest under anaerobic conditions (Xu et al. 2018).

Since the formation of one mole of mevalonate requires three moles of acetyl‐CoA, acetyl‐CoA is a key precursor for mevalonate formation (Figure 1). In steady‐state *E. coli* growing at 0.2 h^−1^, over 62% of the acetyl‐CoA is consumed by citrate synthase coded by the *gltA* gene, while 22% is used for biomass (Zhao et al. 2004). Both ACAT and HMGS must directly compete with citrate synthase and other enzymes for the acetyl‐CoA pool. Unsurprisingly, a simple *gltA* deletion increases mevalonate yield (Satowa et al. 2020). However, a Δ*gltA* strain does not grow on glucose as the sole carbon source (Wu and Eiteman 2016; Tovilla‐Coutiño, Momany, and Eiteman 2020), and therefore, media must be supplemented with glutamate or complex components like yeast extract or peptone.

Given the important role that citrate synthase plays in the availability of acetyl‐CoA, we hypothesize that curtailing, but not eliminating, the activity of citrate synthase (GltA) will increase the availability of acetyl‐CoA toward mevalonate. Modification of citrate synthase by the use of either designed or random point mutations can generate less active enzyme variants (Tovilla‐Coutiño, Momany, and Eiteman 2020; Rajpurohit and Eiteman 2024; Dodelin 2024). Since reducing citrate synthase activity directly diminishes cell growth rate on glucose as the sole carbon source (Tovilla‐Coutiño, Momany, and Eiteman 2020; Dodelin 2024), we expect that increased mevalonate yield could sacrifice mevalonate productivity. Furthermore, incorporating glutamate or other carbon sources into the medium will improve growth, and medium supplementation will become increasingly necessary for strains with the most severe citrate synthase mutations.

The mevalonate pathway also requires NADPH for the reduction of HMG‐CoA to mevalonate (Figure 1). Over 40% of the NADPH required by wild‐type *E. coli* is generated by the pentose phosphate pathway during growth on glucose (Hua et al. 2003), and controlling the partition of carbon between the Embden‐Meyerhof‐Parnas pathway and the pentose phosphate pathway has been previously used to improve the production of NADPH‐dependent biochemicals. For example, hydrogen (H_2_) yield during growth on glucose increased 30% by overexpression of glucose 6P‐dehydrogenase and deletion of phosphofructokinase A encoded by the *pfkA* gene (Sundara Sekar et al. 2016). The formation of butanol, also derived from acetyl‐CoA, improved from 2.7 to 6.1 g/L by the combination of a *pgi* deletion with a 32% reduction in GltA activity by replacing the native promoter (Saini et al. 2016). Increased mevalonate production was also reported by using the non‐oxidative bifido pathway to increase acetyl‐CoA availability in conjunction with the overexpression of glucose 6P‐dehydrogenase and CRISPRi‐based downregulation of the *pfkA* gene (Li et al. 2021). We propose that modification of the native phosphofructokinase (PfkA) could similarly redirect carbon into the pentose phosphate pathway, thereby increasing the availability of NADPH.

In this work, we examine the effect of modifications to citrate synthase and phosphofructokinase on the formation of mevalonate. In both cases, the chromosomal mutations are designed to lead to protein variants with reduced activity of either enzyme. The goal of reduced native GltA activity is to direct more acetyl‐CoA to mevalonate. The goal of reduced phosphofructokinase activity is to direct more glucose‐6P into the pentose phosphate pathway, thereby generating a greater amount of NADPH which might be used for mevalonate formation. We use the medium copy number pMVA1 plasmid (Rugbjerg et al. 2018b; Lutz and Bujard 1997) expressing *atoB, mvaS*, and *mvaE* genes under the control of a constitutive promoter.

## 2 Materials and Methods

### 2.1 Strain Construction

*E. coli* W (ATCC 9637) is the parent strain used in this study (Table 1). The *gltA g*ene deletion was introduced by amplifying via PCR the kanamycin resistance cassette flanked by FRT sites from plasmid pKD4 (Datsenko and Wanner 2000) using PrimeSTAR® Max DNA Polymerase (Takara Bio, Kusatsu, Shiga, Japan) with primers MEP1013 and MEP1014 having 20 bp homology for the kanamycin cassette and ~50 bp homology upstream and downstream of the target *gltA* (see Supporting Information S1: Table 1 for list of all primers). This PCR fragment was transformed into the recipient strain expressing λ‐Red recombinase system plasmid pKD46 (Datsenko and Wanner 2000) using electroporation. After electroporation, samples were allowed to recover at 30°C in Super Optimal Broth with Catabolite Repression (Hanahan 1983) with shaking for 2 h. Successful transformants were selected by growth on Lysogeny Broth (LB) plates containing 40 mg/L kanamycin (LB‐KAN). The plasmid was cured by growth of the resultant strain at 42°C in LB‐KAN, and the kanamycin marker was removed using the flippase containing plasmid pCP20 (Datsenko and Wanner 2000). Each variant strain was additionally created by insertion of a mutant *gltA‐*Kan^R^ fragment amplified by each respective plasmid using primers MEP1096 and MEP1097, with insertion and Kan^R^ removal performed as described above. All strains contained a 102 bp FRT scar immediately after the *gltA* coding region.

**Table 1:** Strains used in this study. Asterisk (*) indicates nucleotide changes to the *gltA* gene that did not lead to an amino acid substitution.

Name	Genotype/Substitutions	Reference
ATCC9637	*E. coli* W	ATCC
DH5α	General *E. coli* cloning strain	New England Biolabs
MEC1353	W Δ*gltA*	This study
MEC1484	W Δ*gltA*::*gltA* ^[Y87N,D101D*,P208L]^	Dodelin (2024)
MEC1501	W Δ*gltA*::*gltA* ^[K167A]^	Stokell et al. (2003)
MEC1502	W Δ*gltA*::*gltA* ^[A267T]^	Tovilla‐Coutiño, Momany, and Eiteman (2020)
MEC1503	W Δ*gltA*::*gltA* ^[F383M]^	Tovilla‐Coutiño, Momany, and Eiteman (2020)
MEC1556	W Δ*gltA*::*gltA* ^[V361A]^	Tovilla‐Coutiño, Momany, and Eiteman (2020)
MEC1557	W Δ*gltA*::*gltA* ^[V361E]^	Tovilla‐Coutiño, Momany, and Eiteman (2020)
MEC1558	W Δ*gltA*::*gltA* ^[P313S]^	Dodelin (2024)
MEC1559	W Δ*gltA*::*gltA* ^[M168L,I252T,P375S,F420F*]^	Dodelin (2024)
MEC1560	W Δ*gltA*::*gltA* ^[T3I,G61A,F121L,L141L*,A233T,F249I,E269E*,E286K,S422G]^	Dodelin (2024)
MEC1377	W Δ*pfkA*	This study
MEC1603	MEC1377 Δ*gltA*::*gltA* ^[K167A]^	This study
MEC1605	MEC1377 Δ*gltA*::*gltA* ^[A267T]^	This study
MEC1606	W Δ*pfkA*::*pfkA* ^[R77A]^‐Kan	This study
MEC1607	MEC1501 Δ*pfkA*::*pfkA* ^[R77A]^‐Kan	This study
MEC1608	MEC1502 Δ*pfkA*::*pfkA* ^[R77A]^‐Kan	This study
MEC1609	W Δ*pfkA*::*pfkA* ^[R171K]^‐Kan	This study
MEC1610	MEC1501 Δ*pfkA*::*pfkA* ^[R171K]^‐Kan	This study
MEC1611	MEC1502 Δ*pfkA*::*pfkA* ^[R171K]^‐Kan	This study
MEC1612	W Δ*pfkA*::*pfkA* ^[R171S]^‐Kan	This study
MEC1613	MEC1501 Δ*pfkA*::*pfkA* ^[R171S]^‐Kan	This study
MEC1614	MEC1502 Δ*pfkA*::*pfkA* ^[R171S]^‐Kan	This study
MEC1615	W Δ*pfkA*::*pfkA* ^[H249F]^‐Kan	This study
MEC1616	MEC1501 Δ*pfkA*::*pfkA* ^[H249F]^‐Kan	This study
MEC1617	MEC1502 Δ*pfkA*::*pfkA* ^[H249F]^‐Kan	This study

The citrate synthase and phosphofructokinase variants used in this study (Tovilla‐Coutiño, Momany, and Eiteman 2020; Dodelin 2024) were introduced into *E. coli* W via a plasmid in four parts. For citrate synthase, the first insert consisted of *gltA* coding region and 570 bp of genomic DNA upstream, amplified by primers MEP1082 and MEP1085. The second insert consisted of the kanamycin resistance cassette flanked by FRT sites from plasmid pKD4 (Datsenko and Wanner 2000), was assembled immediately after the stop codon of *gltA*, and was amplified using primers MEP1084 and MEP1091. The third insert, amplified using MEP1090 and MEP1093, contained 570 bp of the immediate downstream region of *E. coli* W *gltA* and was integrated downstream of the kanamycin/FRT insert. These three fragments were all assembled into the vector pKSI‐1 (Yang et al. 2016), which was amplified using MEP1083 and MEP1092. NEBuilder® HiFi DNA Assembly Kit (New England Biolabs Inc.) was used for the initial assembly of each plasmid and then transformed into *E. coli* DH5α for replication. After purification of each variant plasmid, the *gltA* fragment was amplified using primers that had 20 bp homology with the adjoining fragment.

Phosphofructokinase variant plasmid (pHR‐pfkA) with pKSI‐1 backbone was constructed with fragments including, *pfkA* gene with 500 bp of flanking DNA in upstream region and 78 bp downstream from *E. coli* W, a kanamycin cassette was amplified from pKD4, and 516 bp downstream of *pfkA* gene. All plasmids harboring a single point‐mutated *pfkA* gene were generated from pHR1 using mutagenic primers that incorporated mutations into homologous regions using PCR‐based site mutagenesis. For variant construction, a PCR product containing a variant gene, kanamycin cassette and homology regions greater than 500 bp was amplified from the donor plasmid and integrated chromosomally using pKD46 plasmid (Wu, Tovilla‐Coutiño, and Eiteman 2020). Point‐mutated *pfkA* genes were amplified from the chromosome, gel purified, and sequenced for the confirmed mutations (ACGT, Inc., Wheeling, IL, USA).

### 2.2 Growth Media and Conditions

The defined medium used for shake flask experiments (DGM‐S) contained (per L) 8 g glucose, 3.5 g NH_4_Cl, 0.29 g KH_2_PO_4_, 0.5 g K_2_HPO_4_·3H_2_O, 2 g K_2_SO_4_, 20 mg Na_2_(EDTA)·2H_2_O, 0.45 g MgSO_4_·7H_2_O, 0.25 mg ZnSO_4_·7H_2_O, 0.125 mg CuCl_2_·2H_2_O, 1.25 mg MnSO_4_·H_2_O, 0.875 mg CoCl_2_·6H_2_O, 0.06 mg H_3_BO_3_, 0.25 mg Na_2_MoO_4_·2H_2_O, 5.5 mg FeSO_4_·7H_2_O, 20 mg thiamine·HCl, 50 mg citric acid, and 35.75 g/L HEPES buffer. Chloramphenicol (30 mg/L) was used for plasmid maintenance. Casamino acids (2 g/L) were added to this medium as indicated, and the pH was adjusted to 7.2 with 30% w/v KOH. All cultures were maintained at 37°C.

For shake flask experiments, three colonies of a strain on an LB plate were used to inoculate a 16 × 85 mm tube containing 2 mL DGM‐S, with or without with casamino acids, and grown for 18–24 h at 37°C. These tube cultures were then used to inoculate triplicate 125 mL baffled shake flasks containing 25 mL DGM‐S incubated at 250 rpm. Samples were withdrawn at 24 h for cultures containing casamino acids and at 48 h for cultures grown without casamino acids.

The defined medium used for batch experiments (DGM‐B) contained (per L) 30 g glucose, 8 g NH_4_Cl, 2.4 g KH_2_PO_4_, 2 g K_2_HPO_4_·3H_2_O, 2 g K_2_SO_4_, 20 mg Na_2_(EDTA)·2H_2_O, 0.6 g MgSO_4_·7H_2_O, 0.25 mg ZnSO_4_·7H_2_O, 0.125 mg CuCl_2_·2H_2_O, 1.25 mg MnSO_4_·H_2_O, 0.875 mg CoCl_2_·6H_2_O, 0.06 mg H_3_BO_3_, 0.25 mg Na_2_MoO_4_·2H_2_O, 5.5 mg FeSO_4_·7H_2_O, 20 mg thiamine·HCl, 2.0 g casamino acids, and 50 mg citric acid. Chloramphenicol (30 mg/L) was used for plasmid maintenance, and the pH was adjusted to 7.0 with 30% w/v KOH.

For batch experiments, a single colony was used to inoculate a 16 × 85 mm tube containing 2 mL DGM‐S with casamino acids and grown for 18–24 h at 37°C. A tube culture was used to inoculate a single 250 mL baffled shake flask containing 50 mL DGM‐S with casamino acids, then these cultures were grown to an OD of 0.8 when the culture was transferred to 1.25 L of DGM‐B in a 2.5 L bioreactor (Bioflo 2000, New Brunswick Scientific Co., New Brunswick, NJ, USA). Filtered air supplemented with O_2_ was introduced at 1.25 L/min to maintain the dissolved oxygen above 40%. Agitation was maintained at 400 rpm, pH between 6.8 and 7.0 using 30% KOH. Batch studies were performed in duplicate.

A duplicate set of repeated batch experiments used DGM‐B modified by a reduction of NH_4_Cl to 4 g/L. The fermentations were initiated as batch processes, but when the glucose concentration decreased to about 10 g/L, 40 mL of an 80% w/w glucose solution was added to the culture. During the first supplementation of glucose, trimethylglycine monohydrate (5 mM) was added to the bioreactor.

For a duplicate set of linear fed‐batch experiments, the initial NH_4_Cl concentration of DGM‐B was also 4 g/L, and 40 mL of an 80% w/w glucose solution of glucose was again added to the culture at regular intervals. At the time of that first glucose addition, trimethylglycine monohydrate (5 mM) and other components were also added to increase their respective concentrations by the following amounts (per L): 0.15 g MgSO_4_·7H_2_O, 0.25 mg ZnSO_4_·7H_2_O, 0.125 mg CuCl_2_·2H_2_O, 1.25 mg MnSO_4_·H_2_O, 0.875 mg CoCl_2_·6H_2_O, 0.06 mg H_3_BO_3_, 0.25 mg Na_2_MoO_4_·2H_2_O, 5.5 mg FeSO_4_·7H_2_O, and 50 mg citric acid. At approximately the time of nitrogen depletion from the culture, a solution of 125 g/L NH_4_Cl was continuously added to the bioreactor using a syringe pump at a fixed rate of 660 μL/h (22 mg N/h).

### 2.3 Analytical Methods

Cell growth was measured using optical density (OD) at 600 nm (DU‐650 spectrophotometer, Beckman Instruments, San Jose, CA, USA). Specific growth rate was calculated during by plotting the natural logarithm of OD versus time. Glucose, acetate, and mevalonate concentrations were quantified by HPLC using a Refractive Index detector (Eiteman and Chastain 1997). The mevalonate standard was generated from mevalonolactone dissolved in 1.47% sulfuric acid (Rugbjerg, Feist, and Sommer 2018a).

## 3 Results

One objective of this study was to investigate the effect of diminished citrate synthase activity on the yield and productivity of mevalonate in *E. coli*. A secondary objective was to investigate the effect of diminished phosphofructokinase activity on mevalonate formation. A reduction in citrate synthase activity by modifying the chromosomally expressed enzyme is anticipated to reduce the acetyl‐CoA flux into the TCA cycle. During *E. coli* growth on glucose, citrate synthase variants previously showed a correlation between increased acetate yield and decreased maximum specific growth rate (Tovilla‐Coutiño, Momany, and Eiteman 2020; Dodelin 2024). We hypothesize that mevalonate, a product derived from three molecules of acetyl‐CoA, would show a similar negative correlation: increased severity of a citrate synthase substitution as determined by growth rate would correlate with increased mevalonate yield. This relationship would be further influenced by the added metabolic costs of plasmid maintenance and heterologous protein expression (Silva, Queiroz, and Domingues 2012).

To understand the balance between yield and productivity, we constructed nine citrate synthase variants that previously provided a broad range of growth rates and acetate yields (Table 1). The wild‐type *gltA* strain (W) and the Δ*gltA* strain (MEC1353) represent two ends of the activity spectrum. Four *gltA* variants (MEC1502, MEC1503, MEC1556, and MEC1557) were constructed based on results of a structure‐guided study (Tovilla‐Coutiño, Momany, and Eiteman 2020), and four (MEC1484, MEC1558, MEC1559, and MEC1560) were constructed based on results of random mutagenesis (Dodelin 2024). Additionally, MEC1501 (GltA[K167A]) was constructed because this substitution affects both NADH allostery and acetyl‐CoA binding (Stokell et al. 2003; Lee et al. 2019).

**Figure 2:**
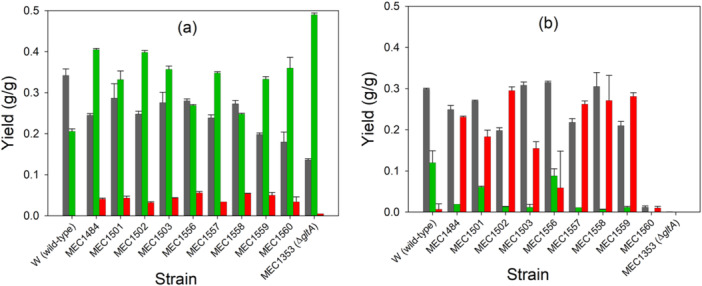
Mevalonate and acetate formation in shake flasks with medium containing 8 g/L glucose (a) with 2 g/L casamino acid and (b) without casamino acids. Biomass yield (gray bars), mevalonate yield (green bars), and acetate yield (red bars) of *E. coli* W strains harboring either the wild‐type *gltA*, *gltA* knockout, or one of nine *gltA* variants. Error bars represent one standard deviation.

Because the *gltA* deletion strain MEC1353 is unable to grow on glucose as a sole carbon source, and some selected citrate synthase variants may similarly have severe growth defects, we first compared mevalonate formation in the presence and absence of “rich” carbon sources through supplementation of casamino acids. Most previous research on mevalonate and isoprenoid formation in *E. coli* have supplemented glucose with other medium components, including casamino acids (Rugbjerg, Feist, and Sommer 2018a; Chatzivasileiou et al. 2019), yeast extract (Wang et al. 2016; Li, Jendresen, and Nielsen 2016), or used highly enriched defined mixtures such as EZ‐Rich medium (Alonso‐Gutierrez et al. 2013; Kim et al. 2015). We therefore first grew the 11 GltA variants transformed with the pMVA1 plasmid in shake flasks in a medium containing 8 g/L glucose and 2 g/L casamino acids. All strains depleted the glucose after 24 h and generated mevalonate (Figure 2a). The W strain expressing the wild‐type citrate synthase attained the greatest biomass yield (Y_X/S_) of 0.342 ± 0.016 g/g and the lowest mevalonate yield of 0.206 ± 0.006 g/g. The citrate synthase knockout strain generated the least biomass (0.136 ± 0.003 g/g) and the greatest mevalonate yield (0.490 ± 0.004 g/g), 90% of the maximum theoretical yield. All variant strains produced intermediate biomass and mevalonate yields, and of the variants, MEC1484/pMVA1 had the greatest mevalonate yield of 0.405 ± 0.003 g/g, approximately two‐fold greater than the wild‐type W strain. Interestingly, W and W Δ*gltA* accumulated virtually no acetate, while all variants generated acetate at yields of 0.03–0.06 g/g.

To understand the role of casamino acids in growth and mevalonate production, we repeated the shake flask experiments, but without the supplementation of casamino acids (Figure 2b). For all cases, the mevalonate yield was much lower during growth without casamino acids than observed in a medium supplemented with casamino acids. For example, the wild‐type W expressing pMVA1 attained a mevalonate yield (Y_M/S_) of only 0.120 ± 0.029 g/g in the absence of casamino acids, 40% less than observed in the presence of casamino acids. No citrate synthase variant strain attained a mevalonate yield greater than 0.10 g/g (Figure 2b). MEC1353/pMVA1 did not grow because of the absence of a functional citrate synthase. MEC1560/pMVA1, the variant attaining the lowest biomass yield of the variant strains with casamino acid supplementation (Figure 2a), grew poorly in the absence of casamino acids and generated no mevalonate (Figure 2b). Also, many of the strains generated significant acetate, with several exceeding an acetate yield of 0.2 g/g (Figure 2b).

**Figure 3:**
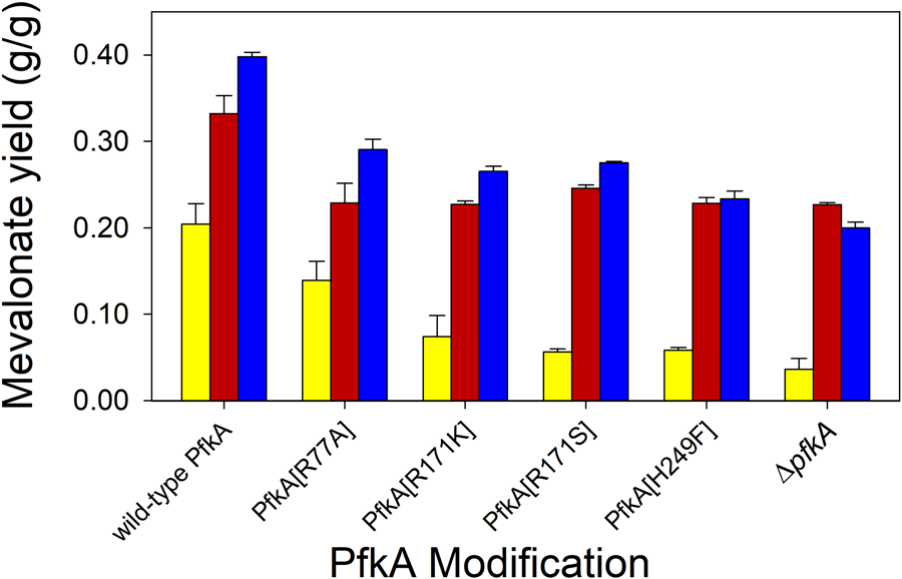
Comparison of phosphofructokinase (PfkA) substitutions on mevalonate yields using glucose with 2 g/L casamino acids in shake flask culture. These PfkA substitutions were incorporated into the wild‐type GltA background (yellow), as well as strains with chromosomally integrated alleles of GltA^[K167A]^ (red) and GltA^[A267T]^ (blue).

A second goal of this study was to examine the effect of modifying phosphofructokinase (*pfkA*). The wild‐type PfkA background was compared with four different *pfkA* variants and the Δ*pfkA* knockout strain. We selected substitutions which were associated with the fructose‐6P binding site. For example, the R77A substitution causes a 2.5× increase in the K_M_ for ATP and a 30% reduction in k_CAT_ compared to the wild‐type (Wang and Kemp 1999). The R171S substitution causes a negligible change in K_M_ and a nearly 75% decrease in k_CAT_ compared to the wild‐type enzyme (Hellinga and Evans 1987), while *E. coli* expressing an enzyme with the R171K substitution shows an 81% greater growth rate than the *E. coli* expressing the enzyme with the R171S substitution (Rajpurohit 2023). The H249F substitution decreased the growth rate by only 60% (Rajpurohit 2023). We combined these four PfkA substitutions and the Δ*pfkA* strain (five strains) with the wild‐type GltA and two GltA substitutions (A267T and K167A), resulting in 15 additional strains. Because the presence of casamino acids appeared important for mevalonate formation (Figure 2ab), we again used strains transformed with the pMVA1 plasmid in shake flasks using a medium containing 8 g/L glucose and 2 g/L casamino acids. Figure 3 shows the mevalonate yield for these shake flask cultures. All PfkA substitutions resulted in lower mevalonate yield compared to the wild‐type PfkA strain. For example, comparing wild‐type GltA strains, the PfkA[F171S] substitution led to 0.05 g/g mevalonate yield, much lower than the 0.20 g/g mevalonate yield observed with the wild‐type PfkA. However, in all cases, both the strain expressing citrate synthase having the A267T or the K167A substitution increased mevalonate yield relative to the wild‐type GltA background. For example, the PfkA[R171S]/GltA[A267T] strain attained a yield of 0.25 g/g, five times greater than the yield observed for PfkA[R171S]/wild‐type GltA strain. Although the effect of casamino acids in the medium is unclear, the conclusion from this study is that the availability of acetyl‐CoA is more important for mevalonate formation than the potential for increased NADPH by directing flux through the pentose‐phosphate pathway by modifying PfkA. For all additional studies, we focused on GltA variants in the wild‐type PfkA background.

**Figure 4:**
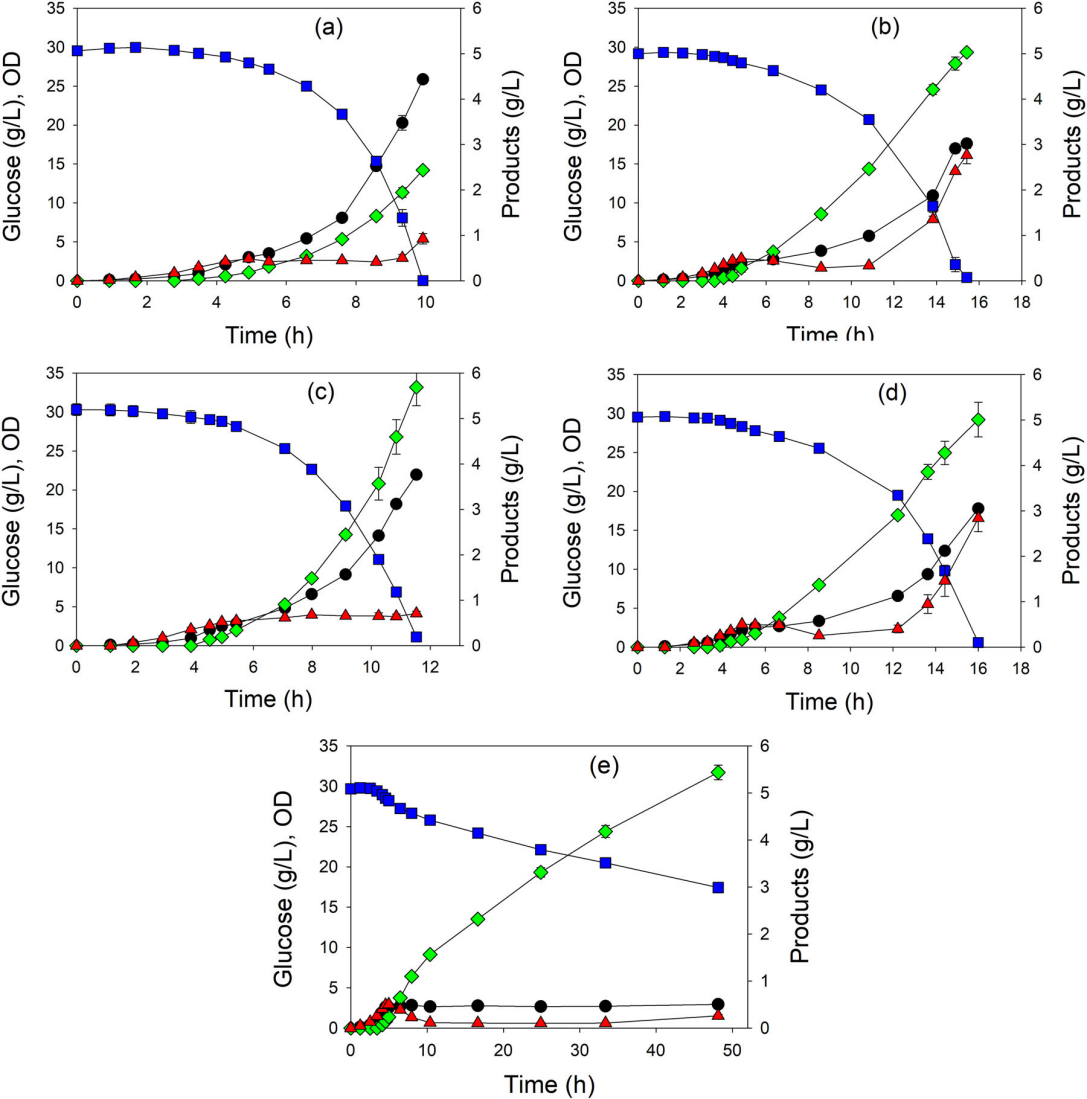
Metabolite concentrations and OD during batch processes using citrate synthase variants: OD (●), glucose (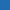), mevalonate (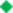), and acetate (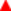). Strains each contained the pMVA1 plasmid: (a) W (wild‐type citrate synthase), (b) MEC1484 (GltA[Y87N D101D* P208L] variant), (c) MEC1501 (GltA[K167A] variant), (d) MEC1559 (GltA[M168L I252T P375S F420F*] variant), and (e) MEC1353 (Δ*gltA*). Strains were grown in duplicate in fully oxygenated 1.25 L bioreactors at a controlled temperature of 37°C and pH of 7 with 30 g/L glucose and 2 g/L casamino acids. Asterisk (*) indicates nucleotide changes to the *gltA* gene that did not lead to an amino acid substitution (Dodelin 2024).

Shake flask conditions generally do not facilitate high cell density and control of pH and oxygenation. We therefore next selected five of the strains expressing the pMVA1 plasmid for controlled batch processes using an initial 30 g/L glucose and 2 g/L casamino acids (Figure 4). The wild‐type strain depleted glucose in about 10 h and generated about 2.5 g/L mevalonate (Figure 4a). MEC1484 (GltA[Y87N D101D* P208L]) required 16 h to consume glucose and generate 5.0 g/L mevalonate (Figure 4b). MEC1501 (GltA[K167A]) depleted glucose within 12 h and generated 5.7 g/L mevalonate (Figure 4c). MEC1559 (GltA[M168L I252T P375S F420F*]) required 16 h to consume glucose and generated 5.0 g/L mevalonate (Figure 4d). Finally, the Δ*gltA* strain MEC1353 consumed only 12 g/L glucose (of the original 30 g/L) by 48 h and had accumulated 5.5 g/L mevalonate (Figure 4e).

Acetate formation also differed among the GltA variants expressing the pMVA1 plasmid. Wild‐type W, MEC1484 and MEC1559 showed an increase in acetate formation at the very end of the processes (Figure 4abd). In contrast, the Δ*gltA* strain MEC1353 depleted acetate that had accumulated during the first 5 h (Figure 4e), while MEC1501 did not generate any additional acetate after the first 5 h (Figure 4c).

**Figure 5:**
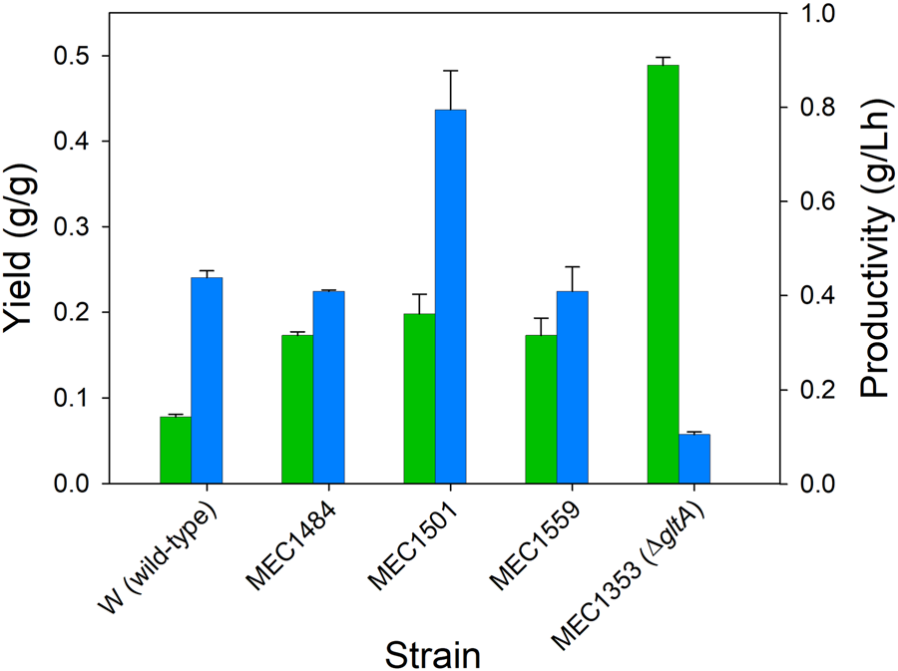
Mevalonate yield (green) and volumetric productivity (blue) during the second phase (“Phase II”) of duplicate batch processes using citrate synthase variants. Phase II commenced for each strain after about 5 h of growth, when a distinct shift occurred between a consistent high growth rate and a lower, strain‐dependent growth rate, likely due to the depletion of a nutrient present in the casamino acids. Strains were grown in fully oxygenated 1.25 L bioreactors at a controlled temperature of 37°C and pH of 7 with 30 g/L glucose and 2 g/L casamino acids.

When the growth was compared among these five strains expressing the pMVA1 plasmid in the bioreactor, two distinct growth phases became evident (Supporting Information S1: Figure 1). During the first approximately 5 h until the OD reached 2.5, all strains attained a specific growth rate of 0.90–0.95 h^−1^. This maximum OD is identical to the maximum attained by the Δ*gltA* strain growing in shake flasks containing 2 g/L casamino acids, suggesting that some key nutrients of the casamino acids had become depleted, which prevents further growth in the absence of citrate synthase activity. During this first growth phase, the performance of the strains was largely indistinguishable, with each by the end of this phase accumulating about 0.2 g/L mevalonate and 0.5 g/L acetate. After this first phase, growth shifted to a second phase displaying a consistent, diminished growth rate which depended on the strain (Supporting Information S1: Figure 1). Specifically, the growth rate of W was 0.46 h^−1^, MEC1501 grew at 0.34 h^−1^, MEC1484 grew at 0.19 h^−1^, and MEC1559 grew at 0.18 h^−1^. The Δ*gltA* strain showed no additional growth. Generally, mevalonate yield and productivity were inversely related during this second phase (Figure 5). For example, wild‐type W expressing pMVA1 attained a mevalonate yield of only 0.082 g/g, although the greater growth rate of this strain (0.46 h^−1^) resulted in a volumetric mevalonate productivity of 0.48 g/L h. In contrast, the Δ*gltA* strain attained the highest yield of 0.49 g/g, but this strain did not grow during the second phase, and thus the volumetric productivity was only 0.12 g/L h. MEC1484 and MEC1559 did not significantly differ, showing twofold greater yield than W, and a slightly lower productivity of 0.45 g/L h. Surprisingly, MEC1501 (GltA[K167A]) attained the greatest yield among the variants (0.20 g/g), but with an intermediate growth rate (0.34 h^−1^), the strain achieved a volumetric productivity of 0.87 g/L h, over 80% greater than the wild‐type W.

The reported calculation for yield excludes the contribution of casamino acids to mevalonate. The approx. 30 g/L glucose contributes 12,800 mg C/L, whereas the 2 g/L casamino acids contribute 490 mg C/L. Although glucose was depleted, the amount of carbon in the casamino acids which was utilized was not determined. Another way to calculate the mevalonate yield is based on total carbon (1 g mevalonate contains 0.49 g C). For example, if we consider that all the casamino acids were consumed by the cells, then the carbon yield for MEC1501 (GltA[K167A]) is 0.21 g C mevalonate/g C. In other words, 21% of the carbon present in the medium was converted to mevalonate.

The rate of glucose uptake can be quantified by the specific glucose uptake rate, whereby the glucose consumption rate is normalized by the cell concentration. For the strain with wild‐type citrate synthase (Figure 4a), the glucose consumption rate during the last 1–2 h of the process was 1.66 ± 0.02 g/g h, while for the Δ*gltA* strain (Figure 4e) this rate was only 0.22 ± 0.01 g/g h. The three variants examined (Figure 4bcd) each showed glucose consumption rates that were statistically indistinguishable at 1.25 ± 0.06 g/g h, despite the significant differences in mevalonate yield.

To optimize mevalonate production further, we sought relatively high volumetric productivity with high yield and therefore selected MEC1501 (GltA[K167A]) as this variant showed both high mevalonate yield and productivity (Figure 5). We furthermore studied MEC1501/pMVA1 under conditions of nitrogen *starvation* by lowering the initial NH_4_Cl (26.2% N) from 8 to 4 g/L. Although the medium also contained casamino acids, the amount of N available in 2 g/L casamino acids is only 154 mg/L, while the amount of N available in 4 g/L NH_4_Cl is 1047 mg/L. Given a biomass yield on nitrogen (Y_X/N_) for *E. coli* of approximately 8 g/g (Rajpurohit and Eiteman 2022), we anticipated a maximum OD of approximately 24, corresponding to a biomass dry weight concentration of about 9.6 g/L. This theoretical maximum OD does not consider some N lost from sampling and the dilution of the culture for pH control. Glucose was maintained by its addition when the concentration decreased below 10 g/L.

**Figure 6:**
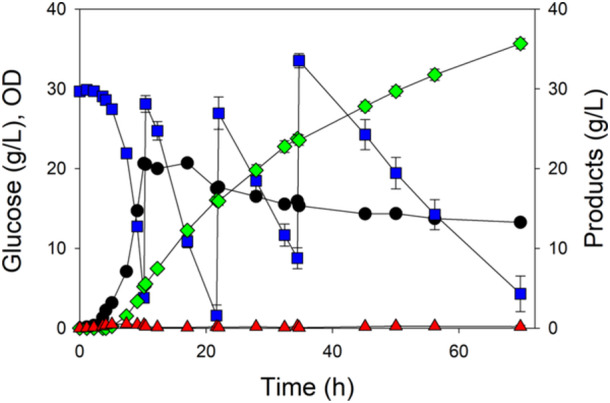
Nitrogen‐starved repeated batch process using MEC1501/pMVA1: OD (●), glucose (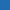), mevalonate (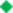), and acetate (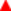). The culture contained 4 g/L NH_4_Cl, sufficient for growth to a cell density (OD) of 21, after which cell growth ceased. Glucose was batch‐wise supplied when its concentration decreased to approximately 10 g/L.

Like the previous batch processes, the growth rate during the initial 5 h exceeded 0.90 h^−1^ until the OD reached 2.5 when presumably one or more key components of casamino acids were depleted, and during the next 5 h, the growth rate reduced to 0.37 h^−1^ (Figure 6). The culture continued to grow to an OD of 20.7 at 10 h, and glucose was added three more times over the course of 70 h. The duplicate processes generated an average of 35.7 g/L mevalonate at an overall yield of 0.37 g/g and productivity of 0.51 g/L h, though the processes slowed during each subsequent glucose addition. For example, during the initial period of glucose consumption and primary cell growth (0–10.2 h) the volumetric productivity was 0.51 g/L h with a yield of 0.21 g/g. During the second phase (10.4–21.6 h) the volumetric productivity and yield increased to 0.94 g/L h and 0.41 g/g, respectively. During the third period of glucose consumption (22.0–34.4 h), the volumetric productivity and yield were 0.63 g/L·h and 0.45 g/g. After the final addition of glucose (34.6–69.7 h), the volumetric productivity decreased to 0.35 g/L·h, while the yield was 0.43 g/g. Furthermore, lacking additional nitrogen for cell growth, the OD decreased by about 35% as time progressed after 10 h.

**Figure 7:**
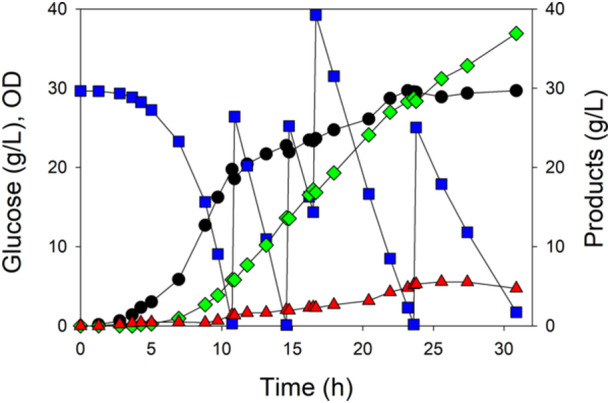
Nitrogen‐limited repeated batch process using MEC1501/pMVA1: OD (●), glucose (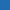), mevalonate (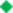), and acetate (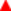). The culture contained 4 g/L NH_4_Cl, sufficient for growth to a cell density (OD) of 21, after which cell growth continued due to the constant feeding of 660 μL/h of a 125 g/L NH_4_Cl solution (22 mg N/h). Glucose was batch‐wise supplied when its concentration decreased to approximately 10 g/L.

With more glucose and less ability to form biomass, the nitrogen‐starved repeated‐batch process achieved about twice the mevalonate yield (0.43 g/g, Figure 6) as the single batch process (0.20 g/g, Figure 5). However, the rate of mevalonate formation decreased appreciably during the process. To retain high yield but improve productivity, we conducted additional fed‐batch experiments with MEC1501 using a continuous linear feed of ammonium designed to achieve a growth rate of less than 0.1 h^−1^ commencing at the time of nitrogen depletion. This nitrogen‐*limited* strategy attained an average of 36.9 g/L in 31 h for an overall volumetric productivity of 1.20 g/L h (Figure 7), more than twice the productivity observed in the repeated‐batch, nitrogen‐starved processes. This increased productivity was accompanied by a 50% increase in cell density and a 25% decrease in the overall yield to 0.31 g/g. In contrast to the nitrogen‐starved processes, acetate accumulation reached 5.5 g/L at 26 h before decreasing to 4.7 g/L.

## 4 Discussion

In this work, we investigated the ability of citrate synthase and phosphofructokinase variants with reduced activity to increase the formation of mevalonate. Citrate synthase and phosphofructokinase represent key junctures in metabolism: citrate synthase controls the carbon flux into the TCA cycle and therefore would impact the pool of acetyl‐CoA, while phosphofructokinase influences the carbon flux into the pentose phosphate pathway, leading to increased potential for NADPH formation. We selected phosphofructokinase for modification instead of phosphoglucose isomerase because the latter enzyme mediates a conversion which is near equilibrium, and a substitution would likely equally affect the rate in both directions. These junctures are relevant to mevalonate formation because the pathway from central metabolism to mevalonate requires three moles of acetyl‐CoA and one mole of NADPH.

We first focused on eight GltA variant strains in comparison to the wild‐type and Δ*gltA* strains with and without casamino acids supplementation. Complex media or media composed of multiple known carbon/energy sources are widely employed to study product formation in metabolically engineered bacteria. Some previous studies have compared production in multiple media. For example, in an *E. coli* strain engineered to produce hydrogen gas, supplementation of glucose with 18 amino acids and four nucleobases increased the concentration of H_2_ synthesized from 10 mM (glucose only) to 16 mM (Mathews, Li, and Wang 2010). Production of 1,3‐butanediol in *E. coli* similarly increased from 48 to 72 mM by the addition of yeast extract (Islam et al. 2023). Mevalonate production by *E. coli* increases 4.4‐fold in a batch culture when a 40 g/L glucose medium was supplemented with 5 g/L yeast extract (Wang et al. 2016), and cinnamic acid titer in *E. coli* was increased by 57% in fed‐batch conditions when the glucose feed was supplemented with casamino acids (Bang et al. 2018). Obviously, there is a preference to avoid medium supplements such as casamino acids because of the cost of materials as well as the potential impact on downstream processing of the product.

Considering the numerous examples of increased product formation using rich medium components, we first focused on eight GltA variants, and compared these strains to the wild‐type and Δ*gltA* strains, to quantify their ability to generate mevalonate with and without casamino acids supplementation. In the initial study using shake flasks (Figure 2ab), we observed a remarkable increase in mevalonate yield in all strains as a result of adding 2 g/L casamino acids. For example, citrate synthase variant MEC1501 generated only 0.02 g/g mevalonate when glucose was the sole carbon source, but 0.41 g/g mevalonate when an additional 2 g/L casamino acid was present. One reason for the increased yield in the presence of casamino acid supplements is the simple fact that the availability of these nutrients permits more glucose to be available for mevalonate. This conclusion would be consistent with reports that supplementation of yeast extract reduces glucose uptake and acetate generation (Han, Lim, and Hong 1992). However, not all of the increase can be attributed exclusively to the greater availability of glucose.

The overexpression of proteins in general greatly impacts the metabolism of *E. coli*, and as a result, energy requirements for the cell are increased due to the formation of the desired enzymes, to greater synthesis of stress‐response proteins, and to higher maintenance energy needs (Özkan et al. 2005). As a result, the requirement for amino acid synthesis increases greatly, and 10 of those required amino acids are derived from the TCA cycle intermediates α‐ketoglutarate and oxaloacetate (Eiteman and Altman 2006, Kaleta et al. 2013). As α‐ketoglutarate and oxaloacetate are removed from the TCA cycle for enzyme formation, these components need to be replaced by anaplerotic routes. Oxaloacetate is replenished by *E. coli* independently of the TCA cycle from phosphoenolpyruvate (PEP) using PEP carboxylase (*ppc*) or PEP carboxykinase (*pck*) (Tan et al. 2013). However, α‐ketoglutarate is generated biochemically from oxaloacetate through citrate, which therefore requires acetyl‐CoA and citrate synthase for its synthesis from glucose (Kaleta et al. 2013). Indeed, glutamate, derived directly from α‐ketoglutarate, is the most abundant amino acid in casamino acids (Jackson and Slininger 1993). Thus, another likely reason for the significant benefit that a casamino acid supplement has to mevalonate yield for citrate synthase variants is explained by the increased availability of amino acids derived from α‐ketoglutarate for protein synthesis specifically in variants having reduced citrate synthase activity.

Metabolic flux is tightly regulated at numerous nodes in *E. coli*. High glucose uptake tends to increase acetate formation (Kleman and Strohl 1994). To avoid inefficient carbon utilization, *E. coli* has evolved mechanisms to reduce glucose uptake. When TCA cycle intermediates oxaloacetate and α‐ketoglutarate reach high concentrations, they inhibit the phosphoenolpyruvate‐carbohydrate phosphotransferase system (PTS) (Zampieri et al. 2019). For example, α‐ketoglutarate binds to PtsI and prevents its phosphorylation by PEP in a competitive fashion (Venditti, Ghirlando, and Clore 2013), causing nearly complete inhibition at only 4 mM (Doucette et al. 2011). Considering that citrate synthase variants would be expected to generate less α‐ketoglutarate, particularly in the absence of glutamate found in casamino acids, lower availability of this biochemical could increase glucose uptake, leading to a greater bottleneck at acetyl‐CoA concentration. The only relief available to acetyl‐CoA build‐up would be towards mevalonate or the overflow metabolite acetate. In the absence of casamino acids, and the diminished ability of *E. coli* to express the plasmid‐derived proteins, acetate was the preferred product (Figure 2b). In contrast, the presence of casamino acids resulted in low acetate formation (Figure 2a). Additional studies to determine glucose uptake and metabolic fluxes of variant strains during growth on glucose and TCA cycle co‐substrates like α‐ketoglutarate or glutamate would clarify the effects of curtailing metabolism into the TCA cycle.

NADPH is required as a redox cofactor for mevalonate synthesis therefore, we investigated phosphofructokinase metabolic node. Previously, strategies such as overexpressing genes of the pentose phosphate pathway (PPP) (Satowa et al. 2020; Li et al. 2021) for NADPH generation along with deletion of citrate synthase (Satowa et al. 2020) to improve acetyl‐CoA availability for mevalonate production have been used. An alternative approach is to fine‐tune the carbon partition between glycolysis and PPP and the partition at acetyl‐CoA. For each of the three GltA variants selected, the wild‐type PfkA strains attained the greatest yields (Figure 3) with no improvement using PfkA variants. The Δ*pfkA* strain additionally expressing the chromosomal GltA[A267T] or GltA[K167A] variant both attained yields equal to the wild‐type GltA/PfkA strain, and approximately 6× greater mevalonate than the Δ*pfkA* strain with wild‐type GltA. A variant GltA is thus able to overcome the reduction in glycolytic flux and acetyl‐CoA availability caused by the *pfkA* knockout. These results suggest that decreasing PfkA activity does not improve mevalonate formation through the prospect of greater NADPH availability via the PPP. Indeed, a reduction in PfkA activity lowers the intracellular acetyl‐CoA concentration (Rajpurohit 2023), which would be detrimental to the formation of an acetyl‐CoA‐derived product such as mevalonate. These results are consistent with previous studies showing that a *pfkA* deletion decreases the formation of acetyl‐CoA‐derived products significantly (Satowa et al. 2020; Wang et al. 2022), but is not consistent with observations in which a Δ*pfkA* strain improved mevalonate‐derived lycopene production (Wang, San, and Bennett 2013).

Citrate synthase variants allow for a tunable balance between yield and productivity when generating an acetyl‐CoA‐derived biochemical such as mevalonate. Other methods such as a metabolic toggle (Soma et al. 2014) or variation of promoter strength (Van Ooyen et al. 2012) also have been studied, and the use of variants complements these other approaches (and can be used in combination with them). Chromosomal modifications do not need additional triggers and likely do not alter the metabolic burden for the cells. Also, reducing the activity of an enzyme which competes with a pathway to a product, in contrast to outright elimination of expression, would still allow for growth, and thus could readily be used in fed‐batch or continuous modes of operations. Wild‐type W and the Δ*gltA* strain MEC1353 serve as two extremes for mevalonate yield, biomass yield and growth rate. In controlled batch studies the W strain completely metabolized 30 g/L glucose in less than 10 h, but its mevalonate yield was only 0.08 g/g. MEC1353 had a mevalonate yield of 0.49 g/g, but it only consumed 12 g glucose in 48 h. The W strain and MEC1353 achieved mevalonate productivities of 0.25 g/L h and 0.11 g/L h, respectively. Each variant examined demonstrated an intermediate level of mevalonate yield, biomass yield, and growth rate. These characteristics led to an optimal volumetric productivity for MEC1501 (GltA[K167A]) at 0.49 g/L h, nearly twofold greater than the wild‐type. The specific optimal citrate synthase variant likely depends on the relative activity of the two competing pathways, as well as a variety of regulatory circuits. Thus, a library of citrate synthase variants could facilitate the identification of the best balance between growth and product yield. The modifications of citrate synthase result in a change in the Michaelis constant (*K*_M_), the turnover (*k*_cat_) or both (Tovilla‐Coutiño, Momany, and Eiteman 2020), as well as the enzyme's allostery and tendency for hexamer formation (Dodelin 2024). For example, purified GltA[A267T] shows a 94% decrease in *k*_cat_ and a 130% increase in *K*_M_ (Tovilla‐Coutiño, Momany, and Eiteman 2020), while GltA[Y87N D101D* P208L] shows a 91% decrease in *k*_cat_, a 2.8‐fold increase in K_M_ and allostery with both oxaloacetate and acetyl‐CoA, with Hill coefficients of 2.0 (Dodelin 2024). Lower *k*_cat_ and greater K_M_ would tend to lower citrate synthase enzyme activity relative to other enzymes which use the same substrate (acetyl‐CoA) including acetoacetyl‐CoA thiolase (coded by the *atoB* gene) and HMG‐CoA synthase (*mvaS*) in the mevalonate pathway (Figure 1).

Nitrogen‐starved and nitrogen‐limited conditions were examined to increase mevalonate yield and productivity using MEC1501. Many studies have explored the physiological consequence of N‐starvation and N‐limitation (summarized in Rajpurohit and Eiteman 2022). For example, N‐starvation depletes glutamine and glutamate while increasing the intracellular concentration of α‐ketoglutarate (Brauer et al. 2006; Boer et al. 2010), resulting in the inhibition of glucose uptake (Doucette et al. 2011), citrate synthase (Pereira et al. 1994) and PEP synthase (Chulavatnatol and Atkinson 1973). N‐limited conditions more than doubles the specific uptake of glucose compared to glucose‐limited conditions (Sauer et al. 1999). N‐limitation also induces genes of the PPP, which promotes the formation of NADPH (Hua et al. 2003). The first strategy employed was to grow MEC1501 to an OD of 21, at which point the free nitrogen (NH_4_ and from casamino acids) in the medium was exhausted, arresting growth. Glucose thereafter would be used only for cell maintenance or converted to carbon products, and during 70 h, cells generated 35.7 g/L mevalonate (Figure 6). This process attained a similar volumetric productivity as the batch process, but the yield increased to 0.37 g/g. Unfortunately, the specific glucose uptake rate decreased by over 50% from 15 to 20 h to 50–60 h (Figure 6, 0.33 g_glucose_/g h compared to 0.15 g_glucose_/g h). *E. coli* likely catabolized the enzymes used for mevalonate synthesis as a response to nitrogen starvation (Masuda, Toya, and Shimizu 2017). We therefore used a process in which the nitrogen source was added slowly to maintain growth at a nominal rate (< 0.1 h^−1^). This fed batch process achieved a much higher volumetric productivity of 1.20 g/L h, but with some glycolytic flux going toward growth and acetate formation, the mevalonate yield decreased to 0.31 g/g compared to the nitrogen‐starved process. Other work has compared S‐, Mg‐ and N‐starvation for mevalonate formation (Masuda, Toya, and Shimizu 2017), and certainly, future studies could focus on various operational modes (both starvation and limitation) in the context of using enzyme variants for the enhanced formation of products derived from acetyl‐CoA.

## Author Contributions

J.K.D., H.R. and M.A.E. designed the study. JK.D. and A.E.R. performed the cultivations, sampling, and analysis associated with citrate synthase. H.R. performed the cultivations, sampling, and analysis associated with phosphofructokinase. J.K.D., H.R. and M.A.E. interpreted the results and wrote the manuscript. M.A.E. was responsible for funding acquisition and project administration. All authors read and approved the final manuscript.

## Conflicts of Interest

The authors declare no conflict of interest.

## Supporting information

Supporting information.

## Data Availability

The data that support the findings of this study are available on request from the corresponding author. The data are not publicly available due to privacy or ethical restrictions.
