# Supplementary material for: Increased Mevalonate Production Using Engineered Citrate Synthase and Phosphofructokinase Variants of Escherichia coli
Source: Biotechnol Bioeng. 2024 Dec 9;122(3):548–60. doi: 10.1002/bit.28902 (PMC11808435; doi:10.1002/bit.28902)
Supplement: Supplementary file 1 — Supporting information. [file BIT-122-548-s001.docx]

**Supplemental Material**

**Increased mevalonate production using engineered citrate synthase and phosphofructose kinase variants of *Escherichia coli***

Jeffrey K. Dodelin, Abigail E. Rose, Hemshikha Rajpurohit, Mark A. Eiteman

**Table S1**. Primers used in this study

| **Name** | **Description** | **Sequence (5′ – 3′)** |
| --- | --- | --- |
| MEP144 | pfkA_F | TACGCATGGGATATGAGGCGGTACAG |
| MEP145 | pfkA_R | GTGACTGACGAATCACCACGTTATCACC |
| MEP243 | C_gltA_5′ | GTGGAAGTATTGACCAATTCATTCGGG |
| MEP328 | gltA-F | ACTACGGGCACAGAGGTTAACTTTC |
| MEP520 | pKSI-plasmid_F | GCCTGGTAGGGATAACAGGGTAATTGCG |
| MEP521 | pKSI-plasmid_R | CGCAATTACCCTGTTATCCCTACCAGGC |
| MEP522 | Kan confirmation_R | CTGCCATCACGAGATTTCGATTCCCTTCCGGCAACAGATTTTATTTTGCATTCCAAAGTTCA |
| MEP645 | KD4-pfkA_F | GAGGTAGTCATGGTGTAGGCTGGAGCTGCTTCCTAAAGGAATCTGCCTTTTTCCGAAATCATTAATACAG |
| MEP646 | KD4-pfkA_R | TTTTTTCGCGCACATATGAATATCCTCCTTAG |
| MEP829 | gltA_R | CACAGTTGACTAAGCGCAGG |
| MEP895 | 5'_DS_pfkA | TAGAACTAGTGGATCCCCCGGGCAATTGCGTCCACGTCAT |
| MEP897 | 5'_UPS_pfkA | CTTGATATCGAATTCCTGCATACGCATGGGATATGAGGCG |
| MEP898 | 3'_UPS_pfkA | TGGACCATGGCTAATTCCCATGATAAGCGAAGCGCATCAG |
| MEP900 | 3'_Kan | CTGATGCGCTTCGCTTATCATGGGAATTAGCCATGGTCCA |
| MEP901 | 5'_Kan | TTGCAGAATTCATGTAGGCCGTGTAGGCTGGAGCTGCTTC |
| MEP903 | 3'_KSI | CGCCTCATATCCCATGCGTATGCAGGAATTCGATATCAAG |
| MEP912 | 5'-pfkA-R171S | GGAAGTGATGGGCAGCTATTGTGGCGATC |
| MEP913 | 3'-pfkA-R171S | CAGATCGCCACAATAGCTGCCCATCACTTCC |
| MEP917 | 3’-pfkA-R171K | CAGATCGCCACAATATTTGCCCATCACTTCCA |
| MEP932 | 5'-pfkA-H249F | CGCAACTGTGCTGGGCTTCATCCAGCGCGGTGGTTC |
| MEP933 | 3'-pfkA-H249F | GAACCACCGCGCTGGATGAAGCCCAGCACAGTTGCG |
| MEP995 | 5’-pfkA-R171K | CGTGGTGGAAGTGATGGGCAAATATTGTGGCGATCT |
| MEP1004 | 5’-pfkA-R77A | GCGCGTTTCCCGGAATTCGCGGACGAGAACATC |
| MEP1005 | 3’-pfkA-R77A | CGCGGATGTTCTCGTCCGCGAATTCCGGGAAAC |
| MEP1013 | KD4-HA-W-gltA-F | TTTAAGTTCCGGCAGTCTTACGTAATAAGGCGCTAAGGAGACCTTAAATGGTGTAGGCTGGAGCTGCTTC |
| MEP1014 | KD4-HA-W-gltA-R | TAAAATATTTACAACTTAGCAATCAACCATTAACGCTTGATATCGCTTTTCATATGAATATCCTCCTTAG |
| MEP1072 | 5'_KSI | CCTATGACGTGGACGCGATTGCCCGGGGGATCCACTAGTT |
| MEP1073 | 3'_DS_pfkA | TAGAACTAGTGGATCCCCCGGGCAATCGCGTCCACGTCAT |
| MEP1082 | pKSI-Up-gltA-F | CTGCGCTCGGTCGTTCGGCTGTCATGCAAAACACTGCTTC |
| MEP1083 | pKSI-Up-glta-R | GAAGCAGTGTTTTGCATGACAGCCGAACGACCGAGCGCAG |
| MEP1084 | gltA-Up-Kan-D-F | AAAGCGATATCAAGCGTTAATGGGAATTAGCCATGGTCCA |
| MEP1085 | gltA-Up-Kan-D-R | TGGACCATGGCTAATTCCCATTAACGCTTGATATCGCTTT |
| MEP1090 | Kan-gltA-W-D-F | GAAGCAGCTCCAGCCTACACTGGTTGATTGCTAAGTTGTA |
| MEP1091 | Kan-gltA-W-D-R | TACAACTTAGCAATCAACCAGTGTAGGCTGGAGCTGCTTC |
| MEP1092 | pKSI-glta-W-D-F | AACTCGGTCAGGTGACCACCATCAGGGGATAACGCAGGAA |
| MEP1093 | pKSI-glta-W-D-R | TTCCTGCGTTATCCCCTGATGGTGGTCACCTGACCGAGTT |
| MEP1096 | gltA-Kan-W-ins-F | CTGCTTCCAGATGCGAAAAC |
| MEP1097 | gltA-Kan-W-ins-R | CCTGACCGAGTTCAACCTCT |
| MEP1167 | WgltA-ins-verf-R | GAATAAAGTATTGGAAATTATAGGGG |


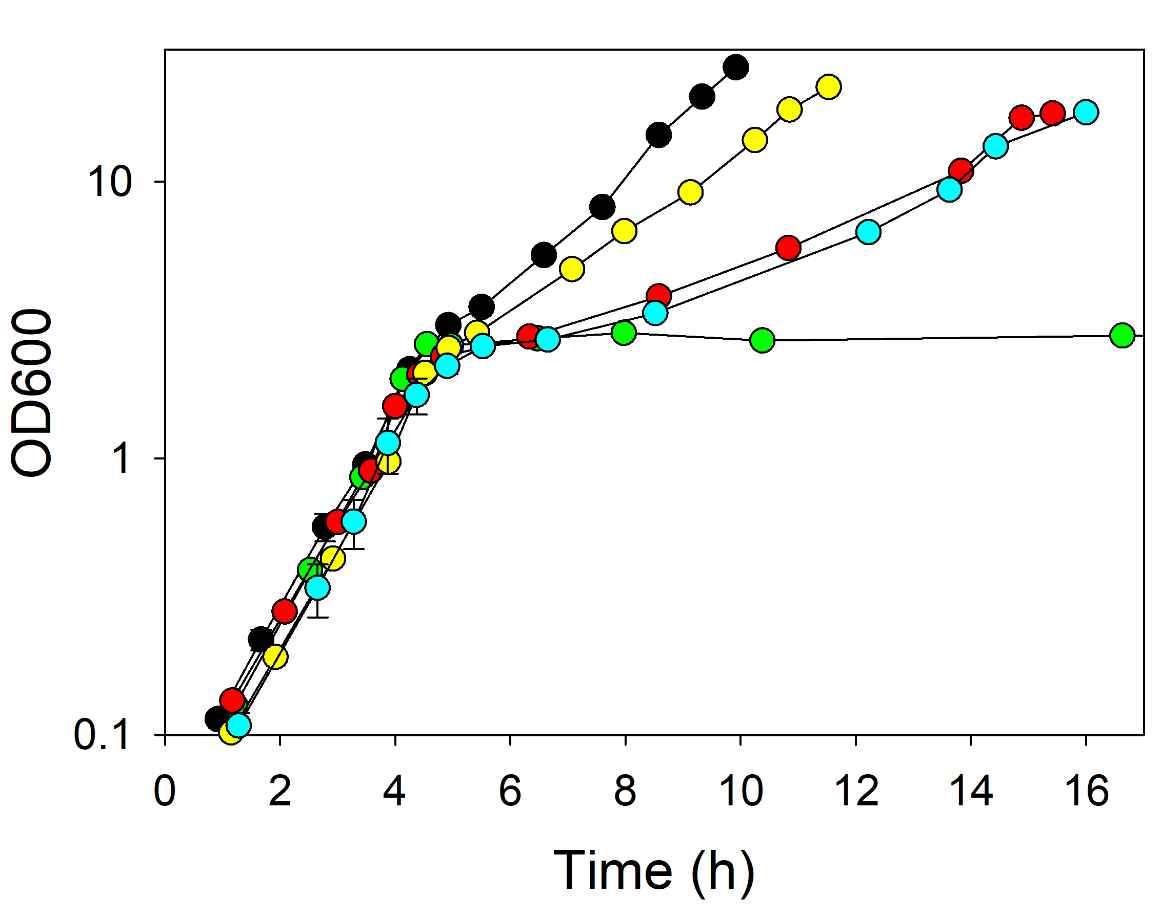


**Figure S1.** Cell density (OD measured at 600 nm) of citrate synthase variants expressing pMVA1 in 1.25 liter batch processes using a medium composed of 30 g/L glucose supplemented with 2 g/L casamino acids as carbon sources. Strains: W (black), MEC1501 (yellow), MEC1484 (), MEC1559 (), MEC1353 (green).
